# The effects of Alpha-2a and Beta-1a Interferons on the Coronavirus disease 2019 prognosis

**DOI:** 10.5339/qmj.2023.sqac.10

**Published:** 2023-11-19

**Authors:** Ali Haghbin, Haniyeh Ghaffari-Nazari, Mohammad Reza Taghavi, Vida Vakili, Sahar Rastgou, Maryam Khoshkhui

**Affiliations:** ^1^Department of Pediatrics, School of Medicine, North Khorasan University of Medical Sciences, Bojnurd, Iran.; ^2^Department of Immunology, School of Medicine, Mashhad University of Medical Sciences, Mashhad, Iran.; ^3^Faculty of Medicine, North Khorasan University of Medical Sciences, Bojnurd, Iran.; ^4^Department of Community and Preventive Medicine, Mashhad University of Medical Sciences, Mashhad, Iran.; ^5^Department of Allergy and Immunology, Mashhad University of Medical Sciences, Mashhad, Iran.; ^6^6Allergy Research Center, Mashhad University of Medical Sciences, Mashhad, Iran. Email: khoshkhuim@mums.ac.ir

## Abstract

Background: The pandemic coronavirus disease 2019 (COVID-19) has been associated with substantial mortality worldwide. Efforts have continued to find an effective treatment for COVID-19. In vitro activity of interferon (IFN) subtypes has been shown against the SARS-CoV and MERS-CoV. Furthermore, the superiority of IFN-**β** over IFN-**α**2b and IFN-**α**2a has been demonstrated in MERS treatment. Early studies showed a low plasma level of IFNs in the peripheral blood or lungs of patients with severe COVID-19. This study assessed the effects of IFN-alpha-2a and -beta-1a on the prognosis of patients with covid-19 infection.

Methods: We conducted a triple-blind randomized clinical trial on adult patients with moderate to severe COVID-19 from April 2021 to June 2021. The patients were diagnosed based on clinical and laboratory findings and randomly assigned into four groups (A, B, C, and D) using the envelope allocation method. Patients in group A received IFN **β**-1a; group B received IFN **β**-1a placebo; group C received IFN **α**-2a, and group D was treated with IFN **α**-2a placebo. All patients concomitantly received the national protocol medications as well.

Results: A total of 95 eligible patients were randomly assigned into groups. National Early Warning Score 2 (NEWS2) index showed significant differences between groups only on the first day of admission (p-value = 0.001). CT scan scores on the first and tenth days slightly improved, although they were not statistically significant. Duration of hospitalization and hospital discharge did not significantly differ among all treated groups (Table 1). Mortality rates showed no significant statistical difference between the groups. However, viral clearance significantly accelerated in the patients receiving IFN **β**-1a or IFN **α**-2a (p < 0.05).

Conclusion: It seems that IFN **α**-2a and IFN **β**-1a are ineffective in treating COVID-19 patients. Further randomized clinical trials with large sample sizes are needed to estimate the effects of IFN **α**-2a or IFN **β**-1a on the outcomes of COVID-19 disease.

## Acknowledgment

The authors would like to thank CinnaGen Co. and Pooyesh Darou Co. for kindly providing CinnoVex® (interferon beta-1a) and Pegaferon® (Peginterferon alfa-2a) drugs.

## Figures and Tables

**Table 1.** The average of hospitalization days.

**Measurement**	**Study groups Min-Max (Day)**	**Mean ± SD (Day)**	**p-value**
Duration of hospitalization	IFN **β**-1a (N=31)	2-17	7.45 ± 3.46	0.673
	IFN **β**-1a placebo (N=25)	3-23	7.88 ± 4.21	
	IFN **α**-2a (N=22)	3-27	7.32 ± 5.19	
	IFN **α**-2a placebo (N=17)	3-10	6.35 ± 2.17	
